# The evolving biology of *Mycobacterium tuberculosis* drug resistance

**DOI:** 10.3389/fcimb.2022.1027394

**Published:** 2022-10-05

**Authors:** Richard M. Jones, Kristin N. Adams, Hassan E. Eldesouky, David R. Sherman

**Affiliations:** Department of Microbiology, University of Washington, Seattle, WA, United States

**Keywords:** tuberculosis, mycobacteria, resistance, tolerance, heterogeneity, antibiotics

## Abstract

Tuberculosis, caused by *Mycobacterium tuberculosis* (Mtb) is an ancient disease that has remained a leading cause of infectious death. Mtb has evolved drug resistance to every antibiotic regimen ever introduced, greatly complicating treatment, lowering rates of cure and menacing TB control in parts of the world. As technology has advanced, our understanding of antimicrobial resistance has improved, and our models of the phenomenon have evolved. In this review, we focus on recent research progress that supports an updated model for the evolution of drug resistance in Mtb. We highlight the contribution of drug tolerance on the path to resistance, and the influence of heterogeneity on tolerance. Resistance is likely to remain an issue for as long as drugs are needed to treat TB. However, with technology driving new insights and careful management of newly developed resources, antimicrobial resistance need not continue to threaten global progress against TB, as it has done for decades.

## Introduction

Tuberculosis (TB), caused by *Mycobacterium tuberculosis* (Mtb), has remained a centrally important cause of morbidity and mortality for centuries, but our understanding of TB disease, what causes it and how to combat it has evolved substantially over that time. In some of the earliest written records of the disease, the ancient Greeks recognized that TB was especially deadly, with the venerable “Father of Medicine” Hippocrates warning other physicians against treating advanced cases because the inevitable bad outcomes would damage the doctor’s reputation (Herzog, 1998). Eventually, the 19^th^ century discovery of the TB bacillus and the 20^th^ century introduction of effective chemotherapies seemed to promise a new era in which TB was tamed if not eliminated (Daniel, 2006; Barberis et al., 2017). However, the emergence of drug-resistant isolates was noted in the very first TB chemotherapy trials, and resistance has appeared whenever a new anti-TB agent is introduced (Crofton and Mitchison, 1948; Gillespie, 2002). With an estimated 1.3 million deaths in 2020 (WHO, 2021b), and given the dramatic change in world population, TB may claim as many total lives today as in the years before TB chemotherapy was available. This dismal situation has several causes, including co-morbidities like HIV and diabetes, and gaps in timely diagnosis and treatment, but drug resistance stands widely recognized as one of the major challenges to effective TB control worldwide.

### The classical model of drug treatment and resistance

**Figure 1:**
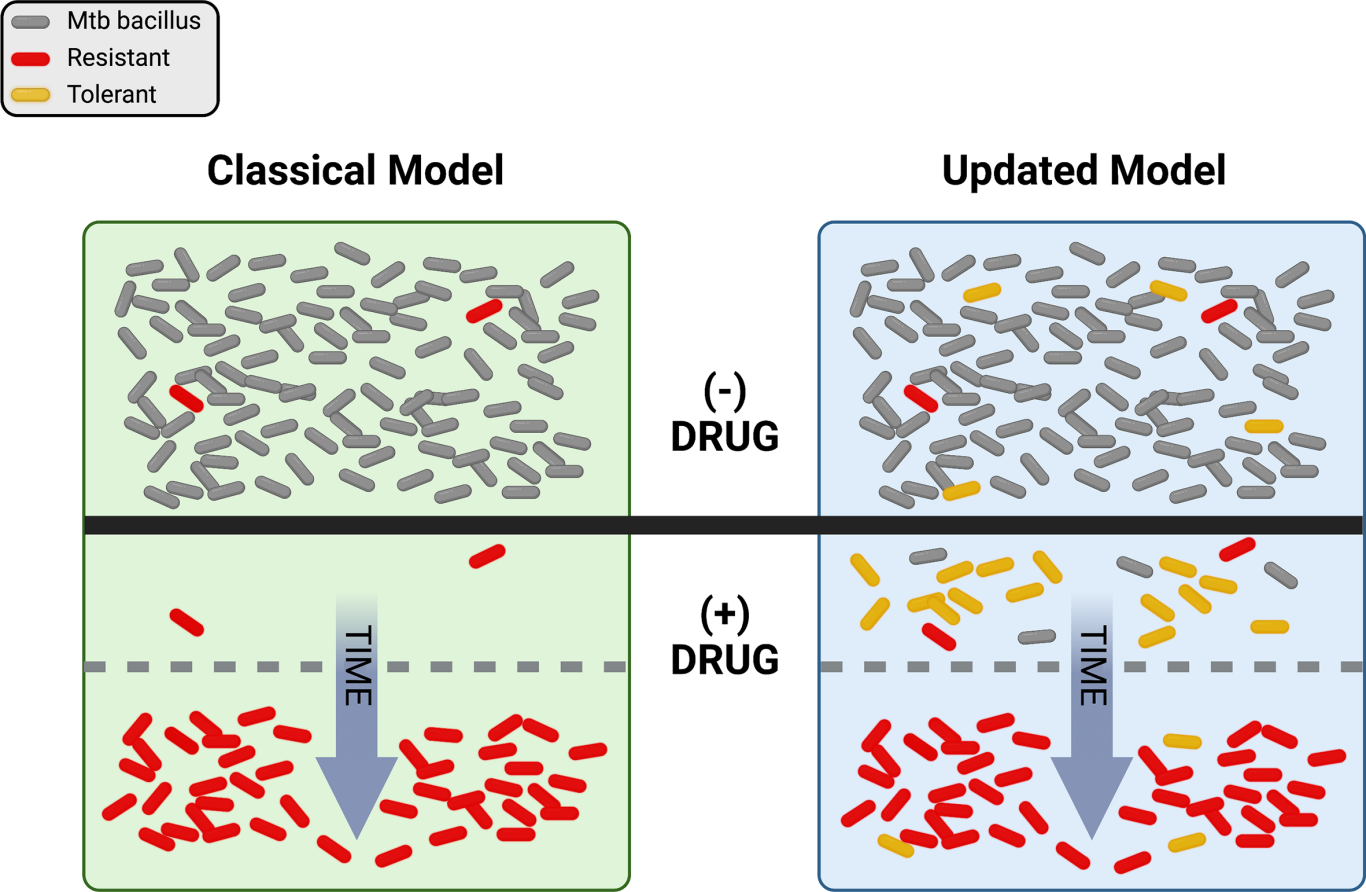
Visual representation of the classical and updated models for development of antibiotic resistance in Mtb. The top portion shows a population of Mtb in the absence of drug selection (- DRUG). In the classical model, pre-existing resistant bacilli (red) in the population grow despite the addition of drug (+ DRUG) and susceptible bacteria (gray) are eliminated over time until only resistant cells remain. In the updated model, pre-existing resistance may still be present, but tolerant bacilli (yellow) may also be present and/or be induced by drug exposure. With drug selection, susceptible bacteria are again eventually eliminated, with resistance dominating the population over time. Figure created with BioRender.com.

Just as ideas about TB have changed, thinking about drug resistance has undergone a significant evolution over time. Antibiotics were initially hailed as “magic bullets”, capable of stopping even lethal infectious diseases in their tracks, but the emergence of resistance-fueled treatment failures led to deeper investigations into the biology of drug response. Driven by powerful advances in genetics and the emergence of molecular biology in the latter half of the 20^th^ century, a concise model of antibiotic action and resistance developed over decades. Briefly, this model proposes that antibiotics work by inhibiting some essential target, generally an enzyme, in the pathogen. Mutations occur at random and exist in each population prior to antibiotic pressure. Resistance emerges when pre-existing mutations promote growth or survival in the presence of the drug (**Figure 1**, left side). A corollary of this model is that resistance is a numbers game. Any pathogen population of sufficient size will harbor at least one mutation conferring resistance to each agent that can be selected by drug exposure, so adding additional drugs to a regimen serves to reduce the rate at which resistance emerges.

Grounded in Darwinian natural selection, this model has long provided a useful framework for exploring antibiotic action and resistance. However, the available data have never all fit comfortably within this paradigm. For example, while current treatment for drug-susceptible TB is ~95% effective, most treatment failures are due to relapses with drug sensitive bacilli (Colangeli et al., 2018). These relapses are hard to explain with the classical model of drug treatment and resistance, since any drug-susceptible bacterium exposed to antibiotics should be eliminated. This phenomenon is not unique to TB – studies with many different bacteria have long revealed curious phenotypes of enhanced survival in the absence of any heritable resistance that are not readily explained by the classical model (Bigger, 1944; Levin and Rozen, 2006). Further, the rate of spontaneous resistance to a three-drug combination has been estimated at greater than 1 in 10^18^ bacteria (Gillespie, 2007), many orders of magnitude greater than the number of TB bacilli in any one patient, possibly more than in all current patients combined. Even considering how some drugs are excluded from some niches *in vivo* (Strydom et al., 2019), it is difficult to reconcile the not-infrequent emergence of widespread resistance to 3 or more agents using the classical model alone. Now, driven again by advances in molecular genetics and especially sequencing, thinking about the evolution of resistance in TB has itself evolved. Here we explore how new observations and insights are promoting an updated model of TB drug resistance, with the promise of new ways to combat the resistance problem.

### Classical resistance determinants in Mtb

Antibiotic resistance is defined as a heritable trait that enables a bacterial population to both survive and replicate in the presence of an otherwise inhibitory antibiotic treatment (**Box 1**). Bacteria can evolve resistance through several well-known mechanisms including alteration of sequence or expression of the target (Ince and Hooper, 2003), alteration of drug modification enzymes (Robicsek et al., 2006), drug efflux (Nikaido, 2009), or gene amplification (Andersson and Hughes, 2009; Sandegren and Andersson, 2009). However, unlike in other bacteria, genetic resistance in Mtb is not known to be associated with horizontal gene transfer and instead is commonly the result of single nucleotide polymorphisms and insertions or deletions (Namouchi et al., 2012; Dookie et al., 2018). This lack of promiscuous gene transfer somewhat simplifies the process of uncovering genetic determinants of drug resistance in Mtb, however complexities remain at every level.

Box 1 **Definitions**

**Antibiotic resistance** is a stably heritable trait that enables a bacterial population to both survive and replicate in the presence of an otherwise inhibitory antibiotic concentration. Mechanisms of antibiotic resistance are tightly associated with mutations in drug targets, activating enzymes, efflux systems, or membrane porins. Of these, mutations in targets and activators are by far most common in Mtb.

**Antibiotic tolerance.** We refer to bacteria that survive high or prolonged concentrations of antibiotics in the absence of stably heritable mechanisms of resistance as drug tolerant persisters. Progeny of tolerant cells exhibit a drug susceptibility profile similar to that of the parental strain. Of note, the nomenclature of drug tolerance can be challenging, as some researchers employ different terms (eg. – phenotypic resistance; non-heritable resistance, antibiotic indifference), and others define tolerance and persistence in more limited ways e.g. (Brauner et al., 2016; Balaban et al., 2019; Urbaniec et al., 2022). In general, these nomenclature distinctions serve to highlight particular subsets of tolerance. Mechanisms of drug tolerance are varied, including reduced metabolic activities, low ATP levels, toxin-antitoxin systems, and stringent response. Note that the definition we employ for drug tolerant persisters is agnostic as to form of tolerance or mechanism.

Resistance to first- and second-line anti-TB drugs is generally associated with known mutations at particular loci. These mutations have been reviewed previously (Almeida Da Silva and Palomino, 2011; Cohen et al., 2019) and are summarized in **Table 1**. Nearly all of these mutations confer resistance through the alteration of a drug target or activator. For example, the target of rifampin (RIF) is the β subunit of bacterial RNA polymerase (RNAP), which is encoded by the *rpoB* gene (Goldstein, 2014). Mutations in *rpoB* have been used to predict resistance to RIF with such a high degree of success (Goldstein, 2014) that an 81-bp region of the *rpoB* gene is now designated the RIF resistance-determining region (Ohno et al., 1996; Ramaswamy and Musser, 1998). However, not all cases of RIF resistance are so straightforward. Distinctions have been drawn between high- and low-level RIF resistance, phenotypes caused by mutations within and outside of the known RIF resistance-determining region of *rpoB* (Shea et al., 2021). Similarly, the presence of mutations in *rpoA* and *rpoC* (Andersson, 2006) as well as secondary site mutations in *rpoB* (Brandis and Hughes, 2013; Meftahi et al., 2016) are now known to play a role in the Mtb RIF resistance profile. Additionally, mutation, and therefore resistance, can be induced under drug pressure. For example, the mutation frequency to RIF was found to increase more than a thousand-fold during 14-days of monotherapy (Kayigire et al., 2017).

**Table 1:** Examples of resistance mutations and compensatory mechanisms in Mtb.

Drug	Major resistance mutation	Compensatory mechanism	References
Rifampin (RIF)	*rpoB*	*rpoA, rpoC*	(Telenti et al., 1993; Ohno et al., 1996; Ramaswamy and Musser, 1998; Comas et al., 2011; Shea et al., 2021)
Pyrazinamide (PZA)	*pncA*	*pnaB2* (epistatic)	(Konno et al., 1967; Scorpio and Zhang, 1996; Muzondiwa et al., 2021)
Para-aminosalicylic acid (PAS)	*thyA*	*thyX–hsdS.1* intergenic region associated, but not shown to be compensatory	(Rengarajan et al., 2004; Zhang et al., 2013; Coll et al., 2018)
Ethambutol (EMB)	*embCAB* operon	*aftA* (Rv3792)	(Alcaide et al., 1997; Telenti et al., 1997; Safi et al., 2013)
Isoniazid (INH)	*katG*, *inhA*	*ahpC* promoter	(Zhang et al., 1992; Heym et al., 1995; Sherman et al., 1996)
Fluoroquinolones (FQ)	*gyrA*	Extragenic *Rv0890c*, Insertions in *glgC* in *Mycobacterium aurum*	(Takiff et al., 1994; Pi et al., 2020)
Bedaquiline (BDQ)	*mmpR (Rv0678), atpE, pepQ*	*atpB*? (suggested)	(Andries et al., 2005; de Jonge et al., 2007; Huitric et al., 2010; Andries et al., 2014; Nieto Ramirez et al., 2020)
Clofazimine (CFZ)	*pepQ, mmpR*	Unknown	(Almeida et al., 2016)
Pretomanid (PA-824)/Delaminid (DLM)	*ddn, fgd1, fbiA, fbiB, fbiC*, and *fbiD*	Unknown	(Haver et al., 2015; Gomez-Gonzalez et al., 2021)
Linezolid (LZD)	*rrl, rplC*	Unknown	(Hillemann et al., 2008; Beckert et al., 2012)
Capreomycin (CAP)	A1408G mutation in 16S rRNA gene (*rrs*)	Increased expression of *tlyA* leading to methylation of C1409	(Maus et al., 2005; Freihofer et al., 2016)
Streptomycin (STR)	*rpsL*, *rrs, gidB*	*rpsD*?*, rpsE*? (proposed)	(Nair et al., 1993; Meier et al., 1994)

The classical model can shed light on most drug resistant Mtb strains circulating today. Indeed, identifying point mutations in specific loci is the basis of highly successful PCR-based tests for Mtb drug resistance (Stevens et al., 2017). Recently, the WHO has catalogued whole genome sequences and drug resistance profiles of 38,215 Mtb clinical strains (WHO, 2021a). This catalogue makes clear the value of the classical resistance model, while also revealing many mutations of unknown mechanism are linked to resistance. Indeed, for every anti-TB agent, there are resistant strains that continue to elude molecular genetic characterization. In addition, the pre-existing mutation model sheds little light on how drug resistance evolves in Mtb. However, in recent years an updated model has emerged that seeks to incorporate older, seemingly anomalous observations with newer, technology-driven insights to explain more completely the global Mtb drug resistance landscape. To take the 18^th^ century writer and polymath Johann Wolfgang von Goethe badly out of context, “tolerance comes of age”.

## The updated model: Tolerance on the pathway to resistance

In 1944, Joseph Bigger described a subpopulation of *Staphylococci* that survived exposure to penicillin without generating heritable resistance. When those cells were cultured in fresh media and then re-exposed to penicillin, they retained the parent strain’s level of susceptibility (Bigger, 1944). Since then, non-heritable survival in the face of antibiotics has been noted in a variety of bacteria exposed to different agents, including Mtb and the phenomenon has been given many names (McCune and Tompsett, 1956; Levin and Rozen, 2006). Here we refer to bacteria that do not harbor stably heritable resistance and yet survive significant antibiotic exposure as drug tolerant persisters (see **Box 1**). Note that our definition is agnostic as to the mechanism(s) by which tolerance occurs.

A possible link between tolerance and the evolution of resistance immediately suggests itself – other things being equal, the longer bacteria survive, the greater the opportunity to mutate to a stably antibiotic resistant state. This reasoning underpins mathematical models that link the two phenomena (Levin-Reisman et al., 2019), and explains why we discuss tolerance in a review about drug resistance. However, it is important to bear in mind that experimental validation for this link is so far limited to a very small number of examples (Levin-Reisman et al., 2017; Sebastian et al., 2017).

### Tolerance and heterogeneity

**Figure 2:**
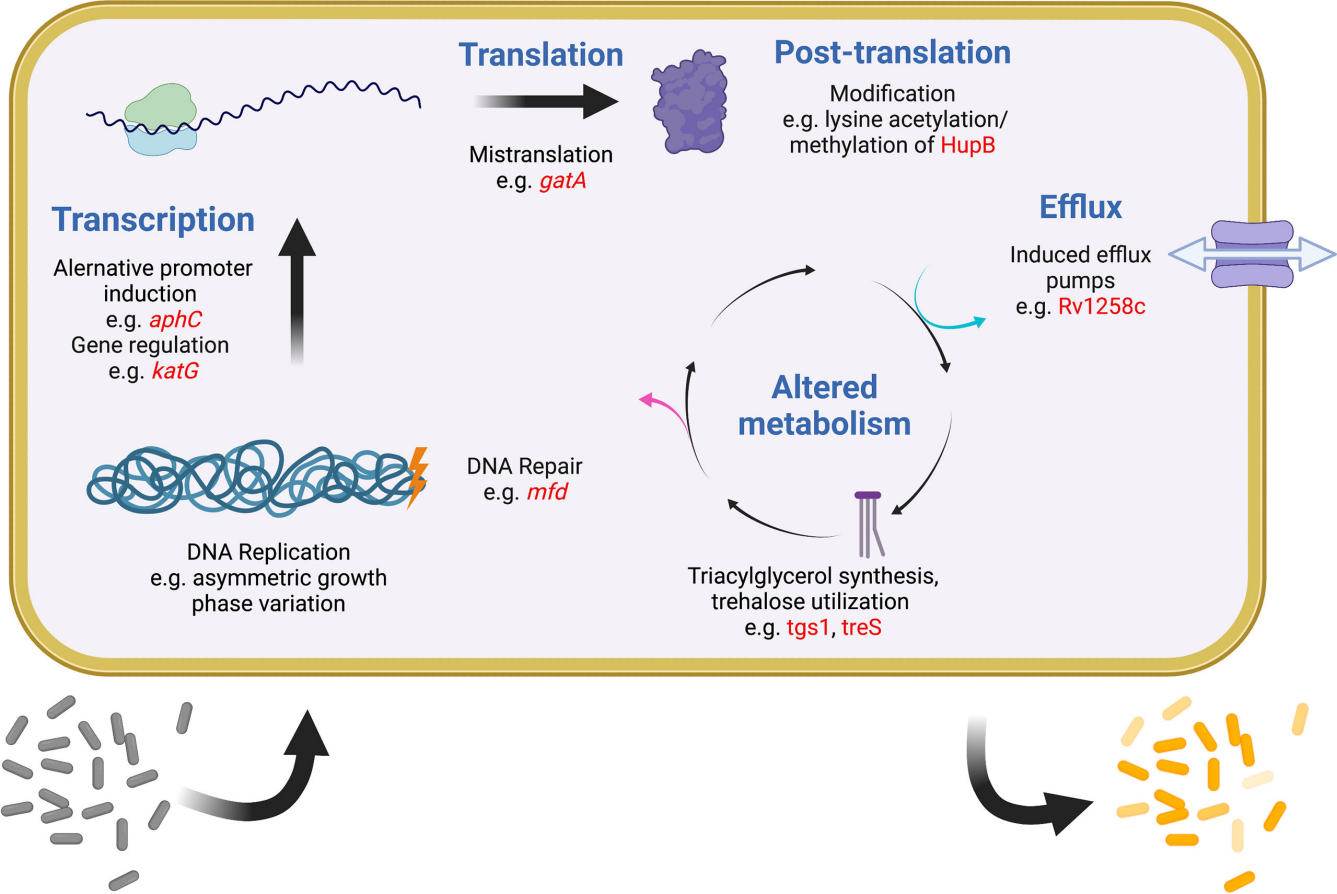
Examples of intracellular mechanisms to generate heterogeneity and tolerance in Mtb. Shown are various cellular processes (blue) at which heterogeneity and tolerance can be generated. Examples of known mechanisms are given at each level with examples in red. One expected outcome of these mechanisms is shown at the bottom, with a sensitive population (gray) becoming differentially tolerant (shades of yellow) after induction of any of these systems. Figure created with BioRender.com.

It was recognized early on that most drugs are less effective on Mtb during infection than they are *in vitro* (McCune and Tompsett, 1956). One important reason is that most antibiotics work best on actively dividing cells, but a robust immune response is one of several mechanisms to slow the Mtb replication rate (Gill et al., 2009; Ford et al., 2011; Colangeli et al., 2014). More recently it has become evident that Mtb has evolved multiple strategies to generate subpopulations of phenotypically distinct bacteria, each with separate growth rates and levels of drug tolerance (Aldridge et al., 2012; Sarathy et al., 2018; Richards et al., 2019). In any given mycobacterial population, variations in replication, DNA repair, transcription, translation, metabolism, and efflux all promote bacterial heterogeneity and are also linked to drug tolerance (Szumowski et al., 2013; Chung et al., 2022). A similar phenomenon is evident within the human lung, where some lesions can support Mtb growth and expand during drug treatment even as other lesions shrink and the patient improves overall (Akira et al., 2000; Xie et al., 2021). Finally, heterogeneity exists among Mtb strains worldwide, driving differences in the accumulation and spread of drug resistance. Recently appreciation has been growing for how widely different mechanisms that promote and maintain bacterial heterogeneity are linked to drug tolerance, treatment failure, and ultimately the evolution of resistance. While this review makes no attempt to be comprehensive, some relevant examples of these mechanisms are provided below (**Figure 2**).

### DNA replication, growth and division

Unlike other bacterial species, mycobacteria elongate from the cell poles, providing a straightforward opportunity for asymmetric growth and division leading to daughter cells with different sizes and growth rates (Aldridge et al., 2012; Santi et al., 2013; Chung et al., 2022). A key member of mycobacterial divisome complex, LamA has been identified as a mediator of asymmetric growth by inhibiting cell-wall synthesis at the new poles (Rego et al., 2017). In the absence of *lamA*, daughter cells are less heterogeneous in size and also display increased sensitivity to RIF and vancomycin compared to wild-type cells (Rego et al., 2017). During *in vitro* growth, Mtb cells shorter in length were found to be more susceptible to RIF, along with being more sensitive to oxidative and nitrite stress (Vijay et al., 2017). Clinically, Mtb with an increased cell length has been associated with host stresses such as iron deficiency and oxidative stress along with an increase in severe pulmonary disease (Vijay et al., 2017). Multidrug-resistant (MDR)-Mtb strains were also found to exhibit increased heterogeneity in cell length compared to drug-sensitive strains during intramacrophage growth and during RIF exposure, which when combined, was further associated with an increase in cell length (Vijay et al., 2017).

In a separate genome-wide association study of clinical Mtb strains, variants in the essential DNA replication initiation factor, *dnaA*, were found to be associated with drug resistance (Hicks et al., 2020). These *dnaA* variants enhance Mtb survival during isoniazid (INH) treatment by interacting with the *Rv0010c-Rv0011c* intergenic region and reduced expression of *katG*, the activator of INH. However, the connection between *Rv0010c-Rv0011c* and altered *katG* expression is not currently understood (Hicks et al., 2020).

### Phase variation

Phase variation is an adaptive mechanism that mediates reversible switching of a gene by genotypic changes, which in turn can lead to reversible or transient drug resistance. Phase variation results from insertions or deletions in a homopolymeric tract (HT) located within the promoter or open reading frame of a gene. Reversible frameshift mutations in HTs are thought to be a result of slipped-strand mispairing errors during replication. Inactivating transient frameshift mutations in the HT of 7 cytosines in the *glpK* gene, which encodes a glycerol-3-kinase, were found to confer heritable drug resistance to INH, RIF, ethambutol (EMB), pyrazinamide (PZA) and moxifloxacin (MOX), but these changes were reversible with additional insertions or deletions in the same HT (Bellerose et al., 2019; Safi et al., 2019). These frameshift mutations were also identified in Mtb-infected mice and in clinical Mtb strains (Bellerose et al., 2019; Safi et al., 2019).

In another example, reversible frameshift mutations in the Mtb *orn* gene in combination with low-level EMB-resistant double *embB-aftA* mutant produced a small colony variant with a significantly higher MIC and further culture led to a rapid reversion of the *orn* frameshift (Safi et al., 2020).

### Genetic regulators mediating tolerance and resistance

Heterogeneity in levels or activity of proteins and flux of metabolites is often facilitated by heterogeneity in gene expression. Recent work has provided examples of transcription factors and regulatory circuits that directly impact drug susceptibility. For instance, bedaquiline (BDQ) is a newer antitubercular drug that inhibits the F_1_F_0_-ATP synthase of Mtb. Network analysis of the Mtb transcriptional response to BDQ suggested that regulators Rv0324 and Rv0880 played important roles in the response to this drug (Peterson et al., 2016). Subsequent work showed that deletion of either Rv0324 or Rv0880 led to improved killing by BDQ but not other drugs. Exposure to capreomycin and MOX also induced Rv0324 suggesting potential antagonism with BDQ, while exposure to pretomanid decreased expression of the Rv0880 BDQ response regulon (Peterson et al., 2016). The addition of pretomanid to novel BDQ-containing regimens was found to improve clearance and reduce relapse in several murine models of TB (Xu et al., 2019), consistent with the synergistic effect of BDQ and pretomanid predicted by the transcriptional response and network analysis.

In another example, a network-based genetic screening approach, the transcriptional regulator-induced phenotype (TRIP) screen, was used to identify regulators that alter susceptibility to INH. Expression of *mce3R*, a TetR family transcription factor, was found to mediate heightened sensitivity to INH (Ma et al., 2021), which was linked to repression of *ctpD*, a gene encoding a metal binding protein (Raimunda et al., 2014; Patel et al., 2016; Ma et al., 2021) not previously known to play a role in INH susceptibility. Disruption of *ctpD* conferred hypersusceptibilty to INH, with increased intracellular accumulation of INH and INH-NAD adduct.

### Drug induced tolerance

Drug exposure has also been shown to induce transcriptional changes conferring tolerance. When exposed to low RIF concentrations, transcription from one promoter is inhibited, allowing increased *rpoB* expression from a second promoter and after a delay, growth can resume despite ongoing drug exposure (Zhu et al., 2018). Subsequent studies have found that the absence of a functional *lepA*, a translation-associated elongation factor, increased RIF tolerance by the upregulation of *rpoB* expression (Wang et al., 2020). Mutations in *lepA* found in clinical Mtb strains were also found to have increased tolerance to RIF (Wang et al., 2020).

### DNA repair

Environmental stresses such as hypoxia, nutrient deprivation, and host immune effectors can generate genome-wide mutations in subsets of cells, leading to heritable genetic resistance (Sebastian et al., 2017; Ragheb et al., 2019; Hicks et al., 2020; Jakkala et al., 2020; Swaminath et al., 2020). The activity and efficiency of different DNA repair pathways influences the rate at which these cells are a reservoir from which resistant isolates emerge.

A recently described link between DNA repair and drug resistance involved the DNA translocase protein Mfd. Mfd has long been thought to initiate nucleotide excision repair at sites of stalled RNAPs, though Mtb cells deficient in Mfd are not more sensitive to DNA-damaging agents. However, Mfd is found to associate with RNAP in the absence of DNA damage and has also been identified as an anti-backtracking factor for RNAP. Interactions of Mfd with the β-subunit of RNAP promote mutagenesis leading to drug resistance in several bacterial pathogens, including Mtb. Deletion of *mfd* led to a decrease in the number of spontaneous resistant mutants to RIF, EMB and ciprofloxacin (Ragheb et al., 2019). Recently, Rv1019, a transcriptional regulator of the TetR family, was found to negatively regulate *mfd* expression. Overexpression of Rv1019 leads to the downregulation of *mfd* and decreased Mtb survival under oxidative stress (Pushparajan et al., 2020). Since Rv1019 is differently expressed during hypoxia and reactivation (Schubert et al., 2015), it would be interesting to know if Rv1019 is the key regulator of Mfd-mediated changes in Mtb leading to drug resistance.

In an *in vitro* persistence model, Mtb was found to develop resistance to RIF or MOX at a much higher frequency than predicted when exposed to continuous lethal concentrations of RIF (Sebastian et al., 2017). Mtb in the RIF persistence-phase was found to carry elevated levels of hydroxyl radicals leading to genome-wide random mutagenesis, generating not only mutants in *rpoB*, but also in *gyrA* demonstrating that exposure to one antibiotic can generate genetic resistance to a different antibiotic (Sebastian et al., 2017). Similarly, exposure to lethal concentrations of MOX also generated high levels of hydroxyl radicals leading to resistance not only to MOX but also to EMB and INH (Swaminath et al., 2020).

### Mistranslation during protein synthesis

Mistranslation happens when an error occurs during the protein synthesis yielding either incorporation of an incorrect amino acid or a truncated protein product. Generally deleterious, mistranslation can be adaptive in the presence of RIF. As noted above, most RIF resistance is due to mutation in a small region of *rpoB*, the RIF resistance determining region (Gagneux et al., 2006b). Increasing the mistranslation rate in *M. smegmatis* led to substitutions of glutamate for glutamine and aspartate for asparagine within the same region, which improved survival during RIF exposure (Javid et al., 2014). This effect is regulated by levels of the GatCAB enzyme complex, where reduced expression results in increased mistranslation and RIF tolerance (Su et al., 2016). Clinical strains with mutations in *gatA*, cause a partial loss of function of the complex along with increased mistranslation and increased RIF tolerance (Su et al., 2016; Li et al., 2021).

### Metabolic regulation

Mtb can utilize a wide variety of carbon sources to support *in vitro* growth. However, Mtb resides *in vivo* within intracellular and extracellular niches where the nutrient composition is thought to be sparse and growth-limiting (Berney and Berney-Meyer, 2017; Sarathy et al., 2018). This slowed growth has traditionally been associated with drug tolerance, as most antibiotics target metabolically active Mtb (Schaefer, 1954). In Mtb, the regulation of several interconnected pathways that control carbon and lipid metabolism contribute to this metabolic slowdown. Rerouting pathways from energy-generation towards energy storage is associated with growth arrest and reduced drug susceptibility. Importantly, even stochastic differences in expression or activity of regulators and rate-limiting steps in these pathways results in bacterial heterogeneity and differential susceptibility to drugs.

For instance, under stress conditions (including drug pressure), Mtb can shift from the growth-promoting TCA cycle to carbon storage in fatty acids *via* triacylglycerol (TAG) synthesis *via* the upregulation of the triglyceride synthase *tgs1* (Sirakova et al., 2006). In the absence of *tgs1*, drug tolerance induced during hypoxia is reversed and Mtb remains sensitive to INH, streptomycin (STR), fluoroquinolones (FQ) and EMB (Baek et al., 2011). The slowing of the TCA cycle alters the turnover of alpha-ketoglutarate, oxaloacetate and reducing agents such as NADH are diminished, resulting in reduced amino acid synthesis and protein translation. Additionally, enzymes such as isocitrate lysases, which are involved in the the glyoxylate bypass, are induced upon exposure to INH, RIF and STR conferring cross-tolerance (Nandakumar et al., 2014). Deficiency in the gluconeogenic enzyme phosphoenolpyruvate carboxykinase encoded by *pckA*, has been implicated in enhanced drug tolerance to INH and BDQ, with this tolerance associated with the overaccumulation of methylcitrate cycle (MCC) intermediates (Quinonez et al., 2022). Similarly, depletion of phosphoenolpyruvate during hypoxia confers tolerance to INH (Lim et al., 2021). In *prpR* mutants, defective regulation of MCC leads to an accumulation of propionyl-CoA conferring tolerance to INH, RIF and ofloxacin (OFX) (Hicks et al., 2018).

In another example, trehalose in Mtb serves as both a carbohydrate store as well as a component of the cell surface glycolipids trehalose monomycolate (TMM) and trehalose dimycolate (TDM). During hypoxia, Mtb has been shown to down-regulate TMM and TDM and channel trehalose into the biosynthesis of central carbon metabolism (CCM) intermediates. In a biofilm model, drug-tolerant persisters were shown to shift trehalose metabolism towards CCM intermediates (Lee et al., 2019). *treS* deletion mutants were unable to shift trehalose metabolism to CMM and showed a rapid depletion of ATP and were also significantly more susceptible to BDQ (Lee et al., 2019).

### Efflux

The Mtb genome encodes a significant number of efflux pumps with a known role in intrinsic and acquired drug resistance, and many of these pumps are also induced during infection (Schnappinger et al., 2003; Rengarajan et al., 2005; Gupta et al., 2010; Szumowski et al., 2013; Pule et al., 2016). Since efflux pumps can have broad substrate specificities, their induction under varying environmental conditions or drug exposure may confer tolerance or resistance to multiple drugs, as is seen with RIF exposure resulting in tolerance to OXF (Louw et al., 2011). Exposure to INH has been shown to induce efflux pumps mediating its tolerance, which then promote the emergence of genetically resistant INH strains (Machado et al., 2012). Additionally, upon infection of macrophages, Mtb has been shown to induce tolerance to numerous antitubercular drugs including INH, RIF, MOX and BDQ that is not tied to reduced growth rate and is sensitive to efflux pump inhibitors such as verapamil (Adams et al., 2011; Adams et al., 2014). Macrophage-induced tolerance to RIF was shown to be mediated by Rv1258c/Tap, an efflux pump also important for intracellular growth (Adams et al., 2011). Further study revealed that strains from all tested global lineages developed macrophage-induced tolerance to RIF except lineage 2 Beijing isolates, which harbor a natural loss-of-function mutation in Rv1258c (Villellas et al., 2013; Adams et al., 2019). In addition, mutations in Rv1258c that were identified in clinical isolates have been linked with resistance to INH, PZA, and STR (Liu et al., 2019).

### Within-host Mtb differences affect drug response

One limitation with the classical model of resistance is that it does not consider within-host variation during TB infection; however, recent studies are beginning to bring evidence of heterogeneity in the Mtb response during drug treatment to light (Borrell and Gagneux, 2009; McGrath et al., 2014). Clinical Mtb strains were collected from patients with delayed culture conversion and WGS was performed. Exposing these strains to RIF *in vitro* revealed drug tolerant variants undetected by bulk WGS-analysis (Genestet et al., 2021). One variant identified by RIF treatment was also enriched during macrophage infection and was found to have a mutation in the gene *mas*, altering its cell surface lipids (Genestet et al., 2021). Characterizing these tolerant sub-populations may help identify patients at risk for treatment failure and the evolution of resistance.

In another study, Mtb isolates from treatment-naïve patients were subjected to WGS to assess within-host diversity. The accumulation of identified mutations varied substantially between isolates from the same individual and were elevated in HIV-negative patients, suggesting that the host immune environment may influence mutation rates (Liu et al., 2020). These results argue that the risk of developing new drug resistance mutations *in vivo* may vary with the host immune environment (Liu et al., 2020). This idea is consistent with evidence from TB patients that host gene signatures exhibiting heightened inflammatory and immune gene expression correlate with longer times to cure and a reduced cure rate (DiNardo et al., 2022).

### Lineage-specific Mtb differences and drug response

It has become clear in the last several years that global variations in Mtb strains have strong impacts on drug response and the evolution of drug resistance. Worldwide Mtb has been separated into seven lineages and many sub-lineages with distinct characteristics that co-evolved with the human populations in which they are present (Brites and Gagneux, 2015). Global lineages differ in their ability to respond to drugs and develop resistance. Members of the modern lineages (2, 3 and 4) are associated with greater disease burden and drug resistance than the ancient lineages, possibly due to an increased spontaneous mutation rate (Borrell and Gagneux, 2009; McGrath et al., 2014). Further, differences *in vitro* and *in vivo*, the genetic background of the strain and the nature of the specific resistance mutation both influence outcomes. For example, there is an association between strain lineage and the type of resistance mutation identified, suggesting that certain Mtb lineages may have characteristics that encourage different routes to resistance (Gagneux et al., 2006a). One study using TnSeq showed that clinically distinct strains have different requirements for *in vitro* growth, including *katG* and *glcB* (Carey et al., 2018). The differences in TnSeq phenotypes of these strains were found to predict their drug resistance rates (Carey et al., 2018).

### Compensatory mutations

Antibiotics, by their nature, target important functions of the bacterial cell. Thus, any mutation that renders a strain resistant has good potential to also reduce the strain’s fitness. This observation once led to the hope that simply reducing the use of antibiotics would lead to fitter, susceptible strains outcompeting resistant ones. However, reduced fitness can also be addressed by compensatory evolution and genetic co-selection (Andersson and Levin, 1999; Andersson, 2006; Andersson and Hughes, 2010).

The fitness cost of a resistance mutation can be measured *in vitro* with isogenic strains serially passaged or grown continuously in chemostats. Such results do not always translate into a host setting, so it is important to also consider how virulence and pathogen transmission are affected. For example, in the 1950s Middlebrook and colleagues found that many INH-resistant Mtb strains were less virulent in Guinea pigs (Middlebrook, 1954; Widelock et al., 1955; Wolinsky et al., 1956). Later, it was revealed that INH-resistant Mtb lacking KatG catalase-peroxidase activity could compensate by overexpressing an alkyl hydroperoxidase (Sherman et al., 1996). Similarly, with regard to RIF resistance, it has been shown that prolonged treatment can result in multidrug resistant strains that have no measurable fitness defect (Gagneux et al., 2006b). These examples illustrate the complex relationship of drug resistance and fitness, where initial costs can be corrected by compensatory mutations that retain the resistance phenotype. It is an important consideration, as such low and no cost mutations have been seen in clinical isolates (Sander et al., 2002). Specific compensatory mutations are shown in **Table 1** and have been reviewed elsewhere (Alame Emane et al., 2021).

One non-canonical form of compensatory mutation that was recently described in Mtb involves restoring fitness of a capreomycin (CAP)-resistant mutant. CAP binds to 16S rRNA and inhibits translation. CAP resistance is conferred by 16S rRNA mutation that also reduces translation efficiency. However, translation can be largely restored by increased expression of an enzyme that methylates a nearby site on the 16S rRNA, significantly reducing the fitness cost of CAP resistance (Freihofer et al., 2016). This is a striking example of a compensatory mutation that relies on changes in expression but acts through post-transcriptional modification. Evidence of these ‘multi-level’ mechanisms of fitness alterations are rare, but it seems likely that more will be discovered and shown to be relevant in other contexts.

## Epistasis

Epistasis refers to a phenomenon where the phenotypic effect of a particular gene allele depends on its genomic background (Hughes and Andersson, 2017). In the context of antibiotic resistance, epistatic interactions between resistance-conferring mutations have a major influence on the fitness of the multidrug-resistant (MDR) isolates and hence their evolution. Epistasis can have either positive or negative outcomes depending on the net effect on bacterial fitness in the absence of antibiotic pressure. Positive epistasis occurs when the fitness cost associated with multiple resistance-conferring mutations is lower than the anticipated additive cost of these mutations if calculated independently. Positive epistasis is a common phenomenon in mycobacteria and in other bacteria as illustrated in numerous studies. For example, a study by Borrel et al. showed that MDR isolates harboring double mutations in *rpoB* H526Y and *gyrA* D94G, conferring resistance to RIF and ofloxacin respectively, were associated with enhanced fitness as opposed to their respective single mutants (Borrell et al., 2013). Similarly, Sun et al. reported a positive epistatic interaction in MDR isolates with double mutations in *rpsL* K43M and *gyrA* D94Y, which confer resistance to STR and fluoroquinolones respectively (Sun et al., 2018). Another example of positive epistasis was also reported by Li et al. where MDR isolates with dual mutations in *rpoB* C531T and *katG* 315C were associated with enhanced fitness (Li et al., 2017). Importantly, those MDR isolates where positive epistasis conferred fitness benefits were associated with better transmissibility and thus were frequently encountered in clinical settings, which supports the idea that positive epistasis plays an important role in the evolution of MDR isolates (Trindade et al., 2009; Borrell et al., 2013).

In addition, positive lineage-specific epistatic interactions were found to be associated with particular Mtb clades. One study identified two epistatic interactions that were exclusively observed in lineage 4 (Coll et al., 2018). Compensatory mutations in *pnaB2* and *thyX–hsdS.1* promoter were found to be associated with resistances to PZA and para-aminosalicylic acid (PAS), due to mutations in *pncA* and *thyA*, respectively.

On the other hand, negative epistasis occurs when the interaction between two or more resistance-conferring mutations aggravates the fitness cost associated with these mutations. For example, FQ resistant isolates with double mutations in *gyrA* and *gyrB* were associated with diminished fitness (Luo et al., 2017). Similarly, in the Borrel et al. study, negative epistasis was observed in MDR isolates with double mutations in *rpoB* H526P and *gyrA* G88C (Borrell et al., 2013). In contrast to positive epistasis, MDR isolates where epistatic interactions resulted in diminished fitness were associated with low transmission rates and were rarely encountered in clinics.

### Epistasis and the evolution of resistance

Several studies have revealed a strong correlation between Mtb lineages and particular drug resistance-conferring mutations, highlighting the major influence of the genetic background on the evolution of drug resistance. For example, one study reported lineage-specific differences in the level of INH resistance due to mutations in *katG* and *inhA* (Fenner et al., 2012). The *katG* mutations were more prevalent in lineage 2 isolates and conferred a high level of INH resistance, whereas *inhA* mutations were more prevalent in lineage 1 and were associated with low levels of INH resistance. Another study found that fluoroquinolone resistance due to mutated *gyrA* occurred more frequently in lineages 2 and 4 (Castro et al., 2020). Similarly, *katG* and *rpoB* mutations occur more frequently in modern Beijing sublineages compared to the ancient strains (Li et al., 2017). These examples demonstrate the major influence the genetic background could have on the evolution of drug response and also may explain why some Mtb lineages, particularly Beijing isolates, are often associated with multidrug resistance (Fenner et al., 2012; Nieto Ramirez et al., 2020; Fursov et al., 2021).

### Epistasis and the level of drug resistance

The classical resistance model relies on using specific genetic determinants to define drug resistance. However, a key limitation of this model is that it tends to ignore the effect of epistasis on the level of drug resistance. Several studies have recently shown that bacterial cells can epistatically exhibit enhanced drug susceptibility despite the presence of a resistance-conferring mutation. For example, a study showed that a loss of function mutation in the *eis* coding region was able to restore amikacin susceptibility in resistant isolates harboring *eis* C-14T mutation (Vargas et al., 2021). Moreover, the same study questioned the validity of *mmpR* mutations as a determinant of bedaquiline and clofazimine resistances if loss of function mutations in *mmpS5* and *mmpL5* were present concomitantly (Vargas et al., 2021).

## Summary and conclusions

So where does drug resistance in Mtb come from, and where is it going? Historically, the classical model (**Figure 1**, left side), in which pre-existing mutations are selected by drug pressure, has proven a very useful framework for our evolving understanding of resistance. However, the updated model (**Figure 1**, right side), with non-obligatory steps through tolerance on the path to resistance, does a better job of describing the rates, types, and patterns of drug resistance within communities and around the world. It is clear that pre-existing mutations conferring resistance do exist in any population of sufficient size, and that resistance does not require a tolerant pre-step. In practice however, with so many different routes to a tolerant state, it is entirely possible that the majority of resistant isolates worldwide today emerged from drug-tolerant precursors.

As described above, tolerance can be stochastic or genetically programmed, and it is frequently linked with the formation and maintenance of heterogeneous sub-populations. Heterogeneity can be recognized at all levels of TB disease, including bacterial subpopulations within individual lesions and across lesions in a single patient, within a single patient over time, within communities, and in different lineages across the globe. In all these cases, we should expect that heterogeneity contributes both to treatment failure and evolution of resistance. Further, since the drug tolerance spawned by heterogeneity is adaptive, we might anticipate that future work will demonstrate that the production of heterogeneous sub-populations is itself under genetic selection and control. In fact, multiple recent reports already point in that direction (Bellerose et al., 2019; Safi et al., 2019; Safi et al., 2020; Ma et al., 2021; Carey et al., 2022; Martini et al., 2022).

The updated model has implications for how we track drug susceptibility and resistance. Increasingly around the world, slow and labor-intensive microbiological drug susceptibility testing is being replaced by faster DNA-based methods, either PCR or next-generation sequencing (NGS) (Dookie et al., 2022; Rowlinson and Musser, 2022). For example, a recent study reported the whole genome sequences of more than 12,000 Mtb clinical isolates, along with sensitivity data for 13 different drugs (The CRyPTIC Consortium, 2022). DNA-based methods offer important advantages in speed, throughput, and safety, as well as altogether novel insights into drug resistance mechanisms (Hicks et al., 2019; The CRyPTIC Consortium, 2022). Catalogues of sequencing results should be widely available and analyzed regularly for potential associations and emerging mutations of interest. However, it is important that these methods are implemented with stringent controls for DNA extraction, sequencing and data handling. NGS sequencing and analysis are not always straightforward (Villellas et al., 2017; Kaniga et al., 2022), and global standards for the application of NGS data to drug susceptibility testing and data reporting are still emerging. Further, there are cases of discordance between whole genome sequencing and drug sensitivity data (Dookie et al., 2022), though these are rare and the extent to which they are due to experimental errors is not yet clear. Finally, NGS generally provides only a snapshot of a bulk sample, without conveying the subtleties of the heterogeneous subpopulations described above. Technologies are in development (**Box 2**) for the identification and characterization of subpopulations, but these are not yet commonplace, and are certainly not yet employed for drug susceptibility testing. Altogether, DNA-based approaches are revolutionizing how we monitor drug susceptibility and resistance and show much promise for further advances but making good on that promise will require both the development of new tools and the rigorous application of those tools in the lab and the clinic.

Box 2 **Technology and our understanding of antibiotic resistance**

As is common in biology, technological advances have been critical in updating our concepts of antibiotic action and the evolution of resistance. Important advances have occurred in:

**Visualization** – Advanced visualization tools such as multiparameter confocal microscopy (Gern et al., 2021; Plumlee et al., 2021) and mass cytometry (Xu et al., 2021) are helping to uncover the complexity of the host response to TB infection. Positron emission tomography (PET) imaging has brought to light the heterogeneity of TB lesions in live animals and humans (Lenaerts et al., 2015). Microfluidics (Molloy et al., 2021) and time-lapse microscopy (Herricks et al., 2020) are revealing the complexity of Mtb populations *in vitro*, and reporter gene technology (Huang et al., 2019) is providing similar insights *in vivo*.

**Next-generation sequencing** – High throughput sequencing has revolutionized to study of drug resistance. With thousands of Mtb genomes sequenced, the diversity of the Mtb pan genome is now evident. Many novel mutations have been linked with resistance to particular drugs, either alone or in association with known resistance loci (Zhang et al., 2013; Zeng et al., 2018; The CRyPTIC Consortium, 2022). Each new mutation must then be studied to see if it truly confers resistance or compensates for fitness defects imposed by mutations at other sites. Single-cell RNA-seq (Pisu et al., 2021) and dual-seq that simultaneously captures transcriptomes of Mtb and host (Pisu et al., 2020) have become important tools to study rare cell types and sub-populations *in vivo*. In addition, next-gen sequencing is central to the updated genetic screens described below.

**Molecular genetics** – Updated approaches in molecular genetics are also shedding new light on antibiotic action and resistance. Tn-seq is not really new technology, but it is being used to gain new insights into resistance mechanisms (Carey et al., 2018). Similarly, CRISPRi screens are identifying new loci associated with resistance to different agents (Li et al., 2022). Also, network-based TRIP screens have been employed to identify novel regulons and effector genes linked to drug sensitivity, tolerance and resistance (Ma et al., 2021).

The updated model also suggests new approaches to combat the emergence of drug resistance. If resistance frequently emerges from tolerant cells, then strategies to kill drug tolerant persisters or restrict their formation should slow the emergence of resistance. While not the topic of this review, eliminating persisters may also shorten the course of current therapy (Chung et al., 2022), so efforts to develop anti-persister therapy should receive high priority. Assays that identify small molecules targeting specific persister subpopulations have been reported (Sukheja et al., 2017; Gold et al., 2021). Hits from these screens could be combined with recent work to identify synergistic drug combinations (Cokol et al., 2017; Katzir et al., 2019; Ma et al., 2019) that can target multiple subpopulations at once. It may also be possible to directly target the machinery that promotes tolerance and resistance. For example, small molecules that inhibit the action of *mfd* (Ragheb et al., 2019) or DNA repair enzymes (Reiche et al., 2017) should reduce the rate at which resistance to other drugs emerge. Also, since small expression changes can have substantial effects on drug tolerance (Ma et al., 2021) and treatment outcome (Colangeli et al., 2018), it should be possible to identify small molecules that specifically alter Mtb gene expression away from tolerance-promoting states. When combined with NGS to characterize individual strains and efforts to uncover host-directed therapies, it is possible to imagine these approaches promoting an era of personalized TB therapy to achieve both improved outcomes and diminished resistance.

In conclusion, the history of drug development argues that resistance will emerge following the introduction of virtually any new agent. However, as the field has evolved and a new model of resistance has emerged, new strategies to protect and preserve agents can be envisioned. With careful monitoring and thoughtful development, we need not tolerate the loss of new agents to TB drug resistance any longer.

## Author contributions

All authors listed have made a substantial, direct and intellectual contribution to the work, and approved it for publication.

## Funding

This review was supported by grants to DS: U19 AI 135976, U19 AI 1625598, and R01 AI 146194. The funders had no role in the decision to publish or prepare this manuscript.

## Acknowledgments

The authors wish to thank David Alland and Shuyi Ma for helpful discussions and suggestions, and Eleanor Lamont for critical reading of the manuscript.

## Conflict of interest

The authors declare that the research was conducted in the absence of any commercial or financial relationships that could be construed as a potential conflict of interest.

## Publisher’s note

All claims expressed in this article are solely those of the authors and do not necessarily represent those of their affiliated organizations, or those of the publisher, the editors and the reviewers. Any product that may be evaluated in this article, or claim that may be made by its manufacturer, is not guaranteed or endorsed by the publisher.
